# Microbial production of hyaluronic acid: current state, challenges, and perspectives

**DOI:** 10.1186/1475-2859-10-99

**Published:** 2011-11-16

**Authors:** Long Liu, Yanfeng Liu, Jianghua Li, Guocheng Du, Jian Chen

**Affiliations:** 1 Key Laboratory of Industrial Biotechnology, Ministry of Education, Jiangnan University, Wuxi 214122, China; 2 School of Biotechnology, Jiangnan University, Wuxi 214122, China; 3 State Key Laboratory of Food Science and Technology, Jiangnan University, Wuxi 214122, China

**Keywords:** Hyaluronic acid, *Streptococcus zooepidemicus*, Metabolic engineering, Molecular weight

## Abstract

Hyaluronic acid (HA) is a natural and linear polymer composed of repeating disaccharide units of β-1, 3-*N*-acetyl glucosamine and β-1, 4-glucuronic acid with a molecular weight up to 6 million Daltons. With excellent viscoelasticity, high moisture retention capacity, and high biocompatibility, HA finds a wide-range of applications in medicine, cosmetics, and nutraceuticals.

Traditionally HA was extracted from rooster combs, and now it is mainly produced via streptococcal fermentation. Recently the production of HA via recombinant systems has received increasing interest due to the avoidance of potential toxins. This work summarizes the research history and current commercial market of HA, and then deeply analyzes the current state of microbial production of HA by *Streptococcus zooepidemicus *and recombinant systems, and finally discusses the challenges facing microbial HA production and proposes several research outlines to meet the challenges.

## Introduction

**Figure 1:**
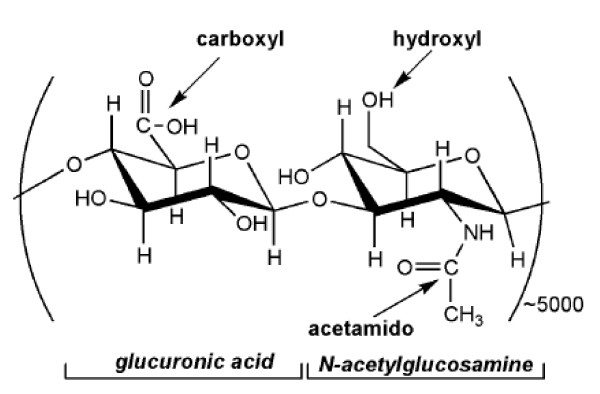
**Structure of disaccharide repeating unit of HA**.

Hyaluronic acid (HA) is composed of disaccharide repeats of *D*-glucuronic acid (GlcUA) and *N*-acetylglucosamine (GlcNAc) joined alternately by β-1, 3 and β-1, 4 glycosidic bonds (Figure 1). The molecular weights of HA from different sources are highly variable, ranging from 10^4 ^to10^7 ^Da. In the human body, HA occurs in the salt hyaluronate form and is found in high concentrations in the skin, umbilical cord, and vitreous humor [1]. HA is also present in the capsules of certain microbial strains (e.g., strains of streptococci). HA possesses significant structural, rheological, physiological, and biological functions. With distinctive moisturising retention ability and viscoelasticity, coupled with its lack of immunogenicity and toxicity, HA finds various applications in the cosmetic, biomedical, and food industries [2].

Traditionally HA was extracted from rooster combs, and now it is mainly produced via microbial fermentation with lower production costs and less environmental pollution [3-7]. HA has been successfully produced on an industrial scale with *Streptococcus *sp. as the main producer. Nevertheless, the production of HA from *Streptococcus *sp. is facing a growing concern due to the fact that streptococci are pathogenic [8]. In this background, the recombinant HA production has attracted an increasing interest, and Novozymes has produced HA with recombinant *Bacillus subtilis *on an industrial scale [8].

In this review, the research history, current HA markets, and HA production by *Streptococcus zooepidemicus *and recombinant systems were summarized. And then the challenges facing the microbial HA production were discussed, and finally several guidelines in the forthcoming research were proposed.

### History

In 1934, Karl Meyer and John Palmer described a new polysaccharide isolated from bovine vitreous humor. They found that the substance contained an uronic acid and an aminosugar, and named the polysaccharide "hyaluronic acid" from hyaloid (vitreous) + uronic acid [9]. The term "hyaluronan" was introduced in 1986 to conform to polysaccharide nomenclature. During the 1930s and 1940s, HA was isolated from many sources such as the vitreous body, umbilical cord, rooster comb, and streptococci [10]. The chemical structure of HA was essentially solved by Karl Meyer and his associates, who found that HA consists of disaccharide repeats of *D*-glucuronic acid (GlcUA) and *N*-acetylglucosamine (GlcNAc) joined alternatively by β-1, 3 and β-1, 4 glycosidic bonds (Figure 1).

The physico-chemical characterization of HA was conducted during the 1950s and 1960s. At a concentration as low as 0.1%, the HA chains were entangled, and this resulted in an extremely high and shear-dependent viscosity [11]. These properties enabled HA to regulate water balance and flow resistance, and also to act as a lubricant, and to stabilize structures [2].

The original development of HA as a product used in clinical medicine was entirely due to Endre Balazs, who developed the first non-inflammatory, highly purified high molecular weight HA from the umbilical cords and rooster combs [12]. In the early 1980s, HA was used to create plastic intraocular lenses for implantation, and it became a major material in ophthalmic surgery. A variety of other applications have since been proposed and developed.

The rooster comb-based extraction process is facing a growing concern over the use of animal-derived components in biomedical and pharmaceutical applications. Hence, microbial fermentation has emerged as a new alternative for HA production. The first commercially fermented HA was produced from *Streptococcus zooepidemicus*, which remains the current common strain in the industrial production of HA [5,6,13]. Nevertheless, the presence of bacterial endotoxins in HA from streptococcal fermentation limits the application of HA in biomedical field [4,8]. Therefore, recombinant HA production has emerged as an attractive alternative. Both Gram-positive and Gram-negative bacteria were used as hosts, including *Bacillus *sp. [3,8], *Lactococcos lactis *[4], *Agrobacterium *sp. [14], and *Escherichia coli *[15].

The applications of HA depend on its molecular weight, which is an important quality parameter for charactering commercial HA products. Yet, the fermentation product is a mixture of HAs with different molecular weights. Obtaining HA with a uniform molecular weight represents a challenge, and much work has been conducted to elucidate the molecular weight control mechanism, which is a current research focus in the field of microbial HA production [16-18].

### HA market

The current worldwide market for HA is estimated to be over $1 billion [2]. With the knee osteoarthritis patient population increasing by 26 percent from 15 million in 2000 to 19 million in 2010, the demand for viscosupplements is expected to escalate. In the US, the first single-injection HA viscosupplementation product, Synvisc-One, was approved in February 2009, and the product gained rapid acceptance by patients and physicians because of its convenience [19]. The European HA viscosupplementation market is shifting toward shorter treatment regimens, and the convenience of undergoing the procedure once will attract more patients through 2013. In the Asia Pacific, the HA viscosupplementation market will be favorably affected by both the aging and physically active demographics, as well as rising awareness of the treatment's benefits among physicians and patients [20].

The global market for dermal fillers is booming, at approximately 759 million USD during 2009, according to Medical Insight Inc. Nowadays, there are almost 100 different dermal fillers on the market, and about half of them are based on HA. American Society for Aesthetic Plastic Surgery reports that about 23, 000 dermatologists, plastic surgeons, and cosmetic surgeons in the US performed more than 11.8 million surgical and non-surgical cosmetic surgery procedures in 2004, generating $12.5 billion in fees. The dermal filler market is expanding at an annual rate of more than 25% through 2011 in the US, and 20% throughout the rest of the world, reaching $1.5 billion in global sales. The launch of Q-Med's Restylane, with NASHA technology (non-animal stabilized HA), has ushered in a new era in dermal enhancement. This filler addresses many of the issues with traditional bovine collagen fillers, namely shelf life, skin testing, and its animal origin.

### Microbial production of HA with *Streptococcus zooepidemicus*

**Figure 2:**
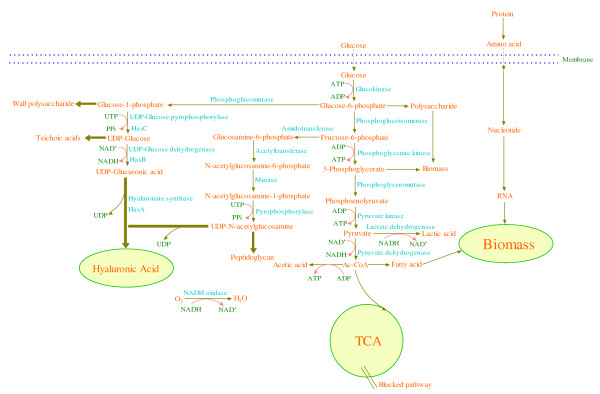
**The biological synthesis pathway of HA in *Streptococcus zooepidemicus***.

Microbial HA production on an industrial scale was firstly achieved in 1980s by Shiseido. The commonly used strain in HA production is *S. zooepidemicus*, which can produce 6~7 g/L HA under the suitable culture conditions. Figure 2 shows the synthesis pathway of HA in *S. zooepidemicus*. However, the following three challenges face the HA production from *S. zooepidemicus*. 1) The broth viscosity reaches as high as 400~500 mPas at 4~5 g/L HA, causing poor mixing and low oxygen mass transfer rate, and thus HA production is severely limited. 2) There exists a strong competition between HA synthesis and cell growth for the common precursors such as UDP-*N*-acetyl-glucosamine and UDP-glucuronic acid. 3) Lactic acid is a main by-product of HA fermentation, and the accumulation of lactic acid results in a strong inhibition of cell growth and HA synthesis. Extensive studies have been conducted to improve HA production by *S. zooepidemicus*, and the recent advances are summarized below.

#### 1) The biosynthesis pathway of HA in *S. zooepidemicus*

The sugar backbone of HA is derived from glucose-6-phosphate and fructose-6-phosphate. The HA synthesis pathway can be divided into two sets. In the first set of reactions, glucose-6-phosphate is converted to glucose-1-phosphate by α-phosphoglucomutase. UDP-glucose pyrophosphorylase adds UTP to glucose-1-phosphate to produce UDP-glucose. Finally, oxidation of the primary alcohol in UDP-glucose by UDP-glucose dehydrogenase yields the first HA precursor, UDP-glucuronic acid. In the second set of reactions, glutamine fructose-6-phosphate amidotransferase transfers the amido group from glutamine to fructose-6-phosphate to yield glucosamine-6-phosphate. Phosphate group rearrangement by phosphoglucosamine mutase produces glucosamine-1-phosphate. The acetylated form of this compound is produced in the next step by phosphoglucosamine acetyltransferase. Finally *N*-acetylglucosamine-1-phosphate pyrophosphorylase activates the intermediate by the addition of UTP thus yielding the second HA precursor, UDP-*N*-acetylglucosamine.

Figure 2 also shows that HA synthesis and cell growth share precursors such as glucose-1-phosphate, UDP-glucose, and UDP-*N*-acetylglucosamine. Thus there is a competition between HA synthesis and cell growth to consume the same precursors, and a high specific growth rate is not favorable for HA synthesis [21]. In addition, it can be seen that the glycolysis and HA synthesis compete for the carbon flux. Therefore, weakening the glycolytic process and reducing the rate of biomass formation are effective for the enhancement of HA titer and molecular weight. For example, HA titer was improved from 5.0 to 6.5 g/L by reducing the biomass formation rate via an intermittent alkaline stress strategy, where the cyclical pH switch from 7.0 (optimal for cell growth) to 8.5 (sub-optimal for cell growth) was done every 2 h during 6-16 h [7]. Both the cyclical temperature switch from 37 to 30°C (reducing cell growth rate) and the addition of pyruvate (weakening the glycolytic process) can significantly increase the HA titer [17].

#### 2) Fermentation medium

Streptococci are nutritionally fastidious lactic acid bacteria and can not synthesize some amino acids [21]. Supplementing some amino acids such as arginine and lysine in the medium was favorable for cell growth and HA production [22]. The chemically defined medium (CDM) containing some nutritional factors essential to growth also can be used for the culture of *S. zooepidemicus*, with the same HA concentration and specific HA production rate relative to complex medium [21]. Zhang et al. developed a serum-free medium with starch as exclusive carbon source, and HA concentration reached 6.7 g/L [23]. The mineral ions and initial glucose concentration also had significant effects on microbial HA production [24,25]. The absence of glucose resulted in a mixed acid metabolism independent of the oxygen supply, while, for the initial glucose concentrations ranging from 5 to 90 g/L, the homolactic metabolism was prevalent [25].

#### 3) Fermentation conditions

Culture conditions (pH, temperature, agitation speed, aeration rate, shear stress, dissolved oxygen, and bioreactor type) significantly influence the microbial HA production. The pH and temperature for HA production by *S. zooepidemicus *were usually at 7.0 and 37°C, respectively [26,27]. The microbial HA production by *S. zooepidemicus *is a typically viscous process, and thus mixing performance and oxygen mass transfer rate significantly influence HA production. The effects of agitation speed, aeration rate, shear stress, and dissolved oxygen on microbial HA production have been extensively studied [22,26-32]. Compared with an anaerobic culture, an aerobic culture gives higher HA titer and molecular weight [21,26]. For example, Armstrong and Johns observed a 20% increase in HA titer when *S. zooepidemicus *were grown under aerobic conditions [21]. Johns et al. also reported that the aerated culture gave higher HA concentration and yield than the equivalent anaerobic fermentation [26]. The stimulation effects of aeration on HA production can be explained by the following: (1) Oxygen may stimulate the HA synthesis as the aggregation of streptococcal cells mediated by their HA capsule shielded them from oxygen metabolites; (2) Dissolved oxygen in the medium can redirect the carbon flux towards lactic acid to acetic acid and thus more ATP can be generated (Y_ATP/glucose _is 3 mol/mol with acetate production against 2 mol/mol with lactate production). The extra ATP generated during the formation of acetate is favorable for the attainment of higher HA titer. (3) The aeration can enhance acetyl-CoA accumulation as such that more acetyl-CoA can be diverged from the central carbon metabolism to replenish acetyl-CoA for the synthesis of HA [32]. Yet, there is a considerable divergence on the impacts of agitation speed and aeration on the microbial HA production. It was observed that HA production was not affected by aeration rate, whereas it decreased with the increase of agitation speed [27]. Hasegawa et al. reported that HA production increased with the increase of aeration rate and agitation speed; nevertheless, too high agitation speed would cause cell damage and led to a drop in HA concentration [33]. The energy status was improved via the overexpression of NADH oxidase in *S. zooepidemicus*, however, little impact on HA titer was observed [6]. This divergence may be explained by the recent study, which indicated that there existed a critical dissolved oxygen level of 5% air saturation for HA synthesis [30]. That was to say, when dissolved oxygen level was lower than 5% of air saturation, the increase of aeration rate and agitation speed was favorable for microbial HA production; and when dissolved oxygen level was higher than 5% of air saturation, there was little impact of agitation speed and aeration rate on HA production.

#### 4) Fermentation mode

Various fermentation modes, such as batch, repeated batch, fed-batch, and continuous culture have been used for HA production [34-41]. Batch culture is the dominant operation mode for HA production. Compared to batch culture, fed-batch culture can shorten fermentation time and thus increase HA productivity [39]. The combination of fed-batch and batch was found to be effective for HA production, namely, *S. zooepidemicus *were cultured in a fed-batch mode with sucrose concentration at 1.0 g/L during 0-8 h and then batch culture was performed during 8-20 h with an initial sucrose concentration of 15 g/L. With this two-stage culture strategy, HA production was increased by 32% compared to the batch culture [38]. Recently, the repeated batch culture has also been employed for HA production, and HA productivity was significantly enhanced [35,36]. In an operation that seeded 31% cell, the volumetric production rate of the repeated batch culture (0.59 g HA/(L·h)) was found to be 2.5-fold of the batch culture (0.24 g HA/(L· h)).

Compared with batch operation, continuous operation can extend culture period, reduce the time spent on reactor turnover, and decrease the polydispersity of molecular weight [21,34]. HA production in a chemostat was, however, not easily achieved due to the instability of the HA-producing phenotype of highly encapsulated streptococci strains at high dilution rate [34]. The highest dilution rate for stable HA production in a chemostat culture was 0.4 h^-1 ^[34]. Therefore, the industrial production of HA cannot be achieved in continuous cultivation [2].

#### 5) Key factors influencing HA molecular weight

Molecular weight is an important quality parameter for a commercial HA product, as it determines the HA's rheological properties, affects physiological response, and defines suitable applications [42,43]. HA with a high molecular weight (greater than 10 kDa) has good viscoelasticity, moisture retention, and mucoadhesion, -- qualities desirable in the areas of ophthalmology, orthopedics, wound healing, and cosmetics. Whereas, HA with a relatively low molecular weight (2-3.5 kDa) or HA oligosaccharides (10-20 sugars in length) have shown to promote angiogenesis, induce expression of inflammatory mediators, and inhibit tumor growth [18].

Compared with anaerobic condition, aeration can increase the molecular weight of HA due to more energy can be produced under aerobic conditions [42]. Moreover, a high dissolved oxygen level favored a high molecular weight, while a high shear stress led to a lower molecular weight [28]. The decrease of HA molecular weight at high shear stress was caused by the reactive oxygen species generated by NADH oxidase. Thus a combination of high dissolved oxygen level and mild shear stress may be an effective strategy to enhance HA molecular weight.

Besides the culture conditions, the balance between the synthesis rate of HA and the providing rate of precursor sugars was also important for the molecular weight. A high ratio of HA synthase gene (*HasA*) to UDP-glucose-6-dehydrogenase gene (*HasB*) resulted in a lower HA molecular weight [18]. Altering this ratio affected the concentration of precursor sugars and ultimately affected the HA size, and it was an effective approach to control HA molecular weight [18]. Of the two sugar precursors, UDP-glucuronic acid and UDP-N-acetylglucosamine, the latter exerted a dominant effect on molecular weight [18]. An overexpression of the genes involved in UDP-glucuronic acid biosynthesis decreased molecular weight; whereas, an overexpression of the genes involved in UDP-*N*-acetylglucosamine biosynthesis increased molecular weight [16]. Thus, manipulating an appropriate balance of UDP-*N*-acetylglucosamine and UDP-glucuronic acid was necessary to obtain HA with high molecular weight. In addition, the balance of glycolytic rate and HA synthesis rate was also important for the molecular weight of HA [17].

### Microbial production of HA with other production systems

Recently, the recombinant HA production has emerged as an attractive alternative that could alleviate safety concerns stemming from pathogenic *S. zooepidemicus *and avian products. Host bacteria, both Gram-positive and Gram-negative, include *Bacillus *sp. [3,8], *L. lactis *[4], *Agrobacterium *sp. [14], and *E. coli *[15,44,45]. An *E. coli *strain (JM109) was engineered into an efficient HA producer by co-expressing the HA synthase from *Pasteurella multocida *and uridine diphosphate (UDP)-glucose dehydrogenase from *E. coli *K5 strain [45]. The engineered strain produced 0.5 g/L HA in shaker flask and 2.0-3.8 g/L HA in a fed-batch culture process in a 1-L bioreactor [45]. *L. lactis *was engineered by introducing the HA synthetic machinery from the *has *operon of *S. zooepidemicus*, and it was found that the insertion of uridine diphosphate-glucose pyrophosphorylase (*hasC*) gene in addition to the HA synthase (*hasA*) and UDP-glucose dehydrogenase (*has B*) genes can significantly increase HA production [46]. The recombinant *L. lactis *NZ9000 strain transformed with the plasmid pSJR3 (co-expressing *hasA*, *hasB*, and *hasC *genes) gave a maximum of 1.8 g/L HA in a 2.4-L batch bioreactor [46]. The *hasA *gene from *S. zooepidemicus *was expressed in *B. subtilis *for the production of HA, and it was found that the production of UDP-glucuronic acid is limiting in *B. subtilis *and that overexpressing the *hasA *gene along with the endogenous *tuaD *gene is sufficient for high-level production of HA in *B. subtilis *[8]. *Agrobacterium *sp. ATCC 31749 was engineered by co-expressing HA synthase gene from *P. multocida*, along with a kfiD gene encoding UDP-glucose dehydrogenase from *E. coli *K5 strain [14]. Coexpression of these two heterologous enzymes enables *Agrobacterium *to produce 0.3 g/L HA in shaker flask cultivation [14]. Table 1 shows the HA production with different strains under different culture conditions. Though HA from Bacillus is commercially available, in general, the recombinant strains produced a lower HA titer than streptococci did, and the forthcoming research should focus on the construction of efficient HA producer with metabolic and genetic tools.

**Table 1:** Overview of HA titer and molecular weight with different microorganisms under different culture conditions

Microorganism	Culture mode	Culture medium	Aeration parameters	HA titer and molecular weight	References
*S. equi *subsp *zooepidemicus *(ATCC 35246)	Batch 2.5 L	Maltose 20 g/L, CDM	600 rpm 1.3 vvm	[HA]: 2.14 g/L MW: 2.1 × 10^6 ^Da	[5]

*S. equi *subsp *zooepidemicus *deficient in *β*-glucuronidase	Batch 100 mL	Glucose 40 g/L, trypton 10 g/L, yeast extract 2.5 g/L	Anaerobic condition	[HA]:0.4430 g/L MW:2.21 × 10^6^Da	[13]

*S. equi *subsp *zooepidemicus *(ATCC 39920)	Batch 3.7 L	CDM	400 rpm 1 vvm	[HA]:3.66 g/L MW: 3.8 × 10^6^Da	[17]

*S. equi *subsp *zooepidemicus *(ATCC 35246)	Batch 2 L	Glucose 60 g/L, CDM	600 rpm 1 vvm	[HA]:4.2 g/L MW: 3.2 × 10^6^Da	[21]

*S. equi *subsp *zooepidemicus *WSH-24	Batch 7 L	Yeast extract 25 g/L, sucrose 70 g/L	200 rpm 0.5 vvm	[HA]:6.7 g/L MW: n.d.	[22]

*S. equi *subsp *zooepidemicus *NJUST01	Batch 500 ml	Starch 50 g/L, glucose 3 g/L, peptone 5 g/L	220 rpm	[HA]:6.7 g/l MW: n.d.	[23]

*S. equi *subsp *zooepidemicus *G1(mutant of ATCC39920)	Batch + pulse 5 L	Glucose 40 g/L, polypeptone 20 g/L, yeast extract 10 g/L	10-80% DO	[HA]: 3.5 g/L MW: 2.19 × 10^6^Da	[28]

*S. equi *subsp *zooepidemicus *(ATCC 35246)	Batch 2 L	Glucose 20 g/L, yeast extract 10 g/L	600 rpm 0.3 vvm	[HA]:2.1 g/L MW:n.d.	[26]

*S. equi *subsp *zooepidemicus *(ATCC 39920)	Batch 3 L	Glucose20 g/L, yeast extract 10 g/L	300 rpm 1 vvm	[HA]:2.3 g/L MW:n.d.	[30]

*S. equi *subsp *zooepidemicus *WSH-24	Batch 7 L	Yeast extract 25 g/L, sucrose 70 g/L	Adding oxygen vector 200 rpm 0.5 vvm	[HA]:6.6 g/L MW:n.d.	[31]

*S. equi *subsp *zooepidemicus *(ATCC 39920)	Fed-batch 2.5 L	glucose 5 g/L, yeast extract 2.5 g/L	20% DO	[HA]:3.5 g/L MW:n.d	[35]

*S. equi *subsp *zooepidemicus *(ATCC 35246)	cC Continuous culture 2 L	Glucose 15 g/L, yeast extract 10 g/L	200 rpm 0 vvm	[HA]:0.6 g/L MW:n.d	[34]

*S. equi *subsp *zooepidemicus *(ATCC 39920)	Repeated batch 3 L	Glucose2 0 g/L, yeast extract 10 g/L, tryptone 1.7 g/L, soytone 0.3 g/L	10%DO	0.59 g HA/(L·h) MW:n.d	[36]

*Streptococcus *sp. ID9102 (KCTC1139BP)	Batch 75 L	Glucose 40 g/L, yeast extract 7.5 g/L, casein peptone 10 g/L	400 rpm 0.5 vvm	[HA]:6.94 g/L MW: 5.9 × 10^6^Da	[37]

*S. equi *subsp *zooepidemicus *WSH-24	Fed-batch 7 L	Yeast extract 25 g/L, sucrose 70 g/L	200 rpm 0.5 vvm	[HA]:6.6 g/L MW:n.d	[38]

*S. equi *subsp *zooepidemicus* (ATCC 35246)	Batch 2 L	Mussel processing wastewater 50 g/L, tuna peptone 8 g/L	500 rpm 0 vvm	[HA]:2.46 g/L MW: 2.5 × 10^6^Da	[39]

*S. equi *subsp *zooepidemicus *(ATCC 35246)	Nitrogen-limited fed-batch 3 L	Yeast extract 10 g/L and a mixture of inorganic salts	600 rpm. 0.05 vvm.	[HA]:2.2 g/L MW:n.d	[40]

*S. equi *subsp *zooepidemicus *(ATCC 39920)	Batch 2 L	Glucose (10-60) g/L, yeast extract 10 g/L	300 rpm, 1.3 vvm.	[HA]:1.8 g/L MW:2.52 × 10^6 ^Da	[41]

*S. equi *subsp *zooepidemicus *(ATCC 39920)	Batch 3 L	glucose 25 g/L yeast extract 60 g/L	250 rpm 2 vvm	[HA]:1.21 g/L MW:4 × 10^7^Da	[25]

*S. equi subsp zooepidemicus *(ATCC 39920)	Batch 125 mL	glucose 25 g/L yeast extract 60 g/L	150 rpm liquid volume: 50 mL in 125 mL	[HA]:0.65 g/L MW:7.4 × 10^7^Da	[24]

*S. equi *subsp *zooepidemicus *mutant	Fed-batch 100 L	CDM	400-1,200 rpm, 0.5-2.0 vvm	[HA]: 6-7 g/L MW:3.2 × 10^6 ^Da	[27]

*S. equi *subsp *zooepidemicus *#104	Batch 2 m^3^	Peptone20 g/L, yeast extract 10 g/L	30 rpm; 0.5 vvm; 0.05 MPa.	MW:4.3 × 10^6 ^Da	[33]

*S. equi *subsp *zooepidemicus *(ATCC) 39920	Batch 250 mL	Agricultural resource derivatives medium	150 rpm	[HA]:0.89 g/L, MW:10^3 ^to 10^4 ^Da	[47]

Recombined *Bacillus subtilis* *(hasA-hasD-VHb)*	Batch 250 mL	Modified minimal medium, 10 g/L glucose	170 rpm liquid volume: 50 mL in 250 mL flask	[HA]:1.8 g/L MW:n.d.	[3]

Recombinant *Lactococcus lactis* *(hasA-hasB)*	Batch 250 mL	M17 medium, 10 g/L glucose	170 rpm liquid volume: 50 mL in 250 mL flask	[HA]:0.65 g/l MW:n.d.	[2]

Recombinant *Bacillus subtilis RB161(hasA-tuaD-gtaB)*	Fed-batch 3 L	minimal medium with sucrose	1300 rpm 1.5 vvm	MW:1 × 10^6 ^Da	[8]

Recombinant *Escherichia coli* *(sshasA-ssugD)*	Fed-batch 250 mL	LB medium	liquid volume: 40 mL in 250 mL flask	[HA]:190 mg/L MW:3.5 × 10^5 ^to 1.9 × 10^6 ^Da	[15]

Recombinant *Agrobacterium *sp. *(pmHas-kfiD)*	Batch 250 mL	LB medium	250 rpm liquid volume: 50 mL in 250 mL flask	[HA]:0.3 g/L MW:0.7 × 10^6 ^to 2 × 10^6 ^Da	[14]

Recombinant *S. equi *subsp *zooepidemicus* *(hasA-hasB-hasC-hasD-hasE)*	Batch 2 L	glucose 20 g/L, uridine 50 mg/L, CDM	300 rpm 0 vvm	MW:1.8 × 10^6 ^to 3.4 × 10^6 ^Da	[16]

Recombinant *Escherichia coli* *(pmHas-kfiD)*	Fed-batch 1 L	Glucose 45 g/L, GlcNAc 11.8 g/L	10%DO	[HA]:3.8 g/L MW:n.d.	[45]

Recombinant *Escherichia coli* (*sz-has*A with rare codon modifications)	Batch	LB medium.	---	[HA]:32.5 mg/L	[44]

Recombinant *Lactococcus lactis* *(hasA*-*hasB*-*hasC*-*hasD*-*hasE)*	Batch 2.4 L	M17 medium, 20 g/L glucose	200 rpm 1 vvm	[HA]:1.8 g/L	[46]

### Perspectives: challenges and opportunities

Though great progresses have been achieved on the microbial production of HA with *S. zooepidemicus *and the recombinant production systems, several challenges remain.

1) The continuous rise in the cost of raw materials weakens the commercial competiveness of microbial HA production, and thus it is necessary to find a cheaper substrate replacement to reduce production cost. Furthermore, the needs of a sustainable society point to the conversion of renewable resources such as agricultural derivatives into valuable bioproducts. Thus, exploring the feasibility of producing HA with cheap crude materials or wastes from the other industrial processes is worth investigating. Mussel processing wastewater (MPW) and tuna peptone (TP) from viscera residue are used for HA production by *S. zooepidemicus*, and the economic analysis indicated that the production cost can be reduced by more than 30% with the by-products as the culture medium [39]. The agricultural resource derivatives such as cashew apple juice was a promising medium for the microbial HA production [47]. For another example, the large amount of crude glycerol produced in the biodiesel industry, if not properly treated, pose a significant environmental concern. Therefore, we can explore the potential of microbial HA production with the crude glycerol as a substrate. Of course, process engineering for the efficient treatment of crude material and metabolic engineering of microbes for the efficient utilization of raw substrates should be considered to achieve this objective.

2) Whether for *S. zooepidemicus *or the recombinant systems like *E. coli*, *B. subtilis*, and *L. lactis*, the key factors limiting HA synthesis need to be further clarified. The tools of metabolic engineering, such as metabolic flux analysis (MFA) and metabolic control analysis (MCA), can be employed to develop a rational strategy to improve HA yield and molecular weight. MFA is an analysis technique used to calculate and analyze the flux distribution of the entire biochemical reaction network during a process. MCA quantifies the relation between genetic modifications or environmental changes and cellular process responses [48]. MCA introduces the control coefficients to quantify the fractional change of cellular output, such as metabolite concentrations and metabolic fluxes, in response to fractional change of system parameters, such as enzyme activities and growth conditions [49]. The combination of MFA and MCA can be used to investigate the metabolic responses of HA producer to the environmental changes or the expression of key genes related with HA synthesis. With the information gathered from MFA and MCA, the optimal strategies (both process control and key genes expression) can be determined to improve HA titer and molecular weight.

3) It is necessary to obtain specially designated molecular weight or uniform size-defined HA to extend the applications of HA and make better HA containing biomedical products. To achieve low polydispersity, we must know the regulatory mechanisms of initiation and elongation during the HA polymer synthesis process. Despite HA polymerization model has been put forward and some key intracellular metabolites influencing molecular weight have been clarified, much work needs to be performed to understand the mechanism of molecular weight control.

## Competing interests

The authors declare that they have no competing interests.

## Authors' contributions

All authors contributed to the background research and writing of the article, as well as the editing. In addition, all authors have read and approved the final version of this manuscript.
